# A cross-sectional assessment of primary healthcare facilities for provision of antenatal care: calling for improvements in Basic Health Units in Punjab, Pakistan

**DOI:** 10.1186/s12961-015-0046-3

**Published:** 2015-11-25

**Authors:** Muhammad Ashraf Majrooh, Seema Hasnain, Javaid Akram, Arif Siddiqui

**Affiliations:** Professor & HOD Community Medicine, Services Institute of Medical Sciences (SIMS) Lahore, 34-B, Jatala House, Atchison Housing Society Raiwind Road, Lahore, Pakistan; Allama Iqbal Medical College, Lahore, Pakistan

**Keywords:** Antenatal care, Basic health units, Cross-sectional, Infrastructure, Primary healthcare facilities, Rural health centres

## Abstract

**Background:**

There are two types of barriers to the utilisation of maternal health and antenatal care (ANC) services, including the supply-side barriers operating at the health facility level and demand-side, affecting the utilisation ANC services by pregnant women. The purpose of the study was to assess the essential resources required for the provision of ANC services in primary healthcare facilities in Punjab, Pakistan.

**Methods:**

A cross-sectional facility assessment was conducted in primary healthcare facilities across Punjab. A multi-stage sampling was used to randomly select nine districts from three stratifications and 19 primary healthcare facilities in the public sector (17 Basic Health Units (BHUs) and two Rural Health Centres (RHCs)) from each district. A total of 171 health facilities were included. Data on infrastructure and availability of equipment, essential supplies, medicines, treatment protocols, and infection control items was collected through pre-tested, semi-structured questionnaires. Univariate analysis was carried out to describe the frequency and percentages of facilities across three ratings (good, average, and poor) by type of facility.

**Results:**

Overall, 28% of facilities had poor infrastructure and the availability of equipment was poor in 16% of the health facilities. Essential supply items, such as urine strips for albumin, blood sugar testing strips, and haemoglobin reagents, were particularly poorly stocked. However, infrastructure and the availability of equipment and supplies were generally better in RHCs compared to BHUs.

**Conclusion:**

Health facilities lacked the resources required to provide quality ANC services, particularly in terms of infrastructure, equipment, supply items, and medicines. The availability of these resources needs to be urgently addressed.

## Background

Improving maternal health is one of the eight Millennium Development Goals, with two specific targets focusing on the reduction of the maternal mortality ratio and achieving universal access to reproductive health, including antenatal care (ANC). The most recent maternal mortality ratio for Pakistan was 276 deaths per 100,000 live births [1]. Further, the proportion of pregnant women who have at least one ANC visit during their pregnancy is 76%, with the proportion of pregnant women having at least four visits decreasing to 37% [2]. ANC coverage (for one ANC visit) in the Punjab province of Pakistan was estimated at 53% for 2 years in the 2007–2008 MICS survey [3]. Similarly to the rest of the country, inequity exists between rural and urban areas: coverage is 50% in rural areas in comparison to 71% in urban areas.

ANC, a core component of maternal and newborn health services, provides opportunities to reach pregnant women with a number of high-efficacy interventions. WHO recommends a minimum of four ANC visits [4] and has specific guidelines on the content of ANC, which should include blood pressure measurement, urine testing for bacteriuria and proteinuria, blood testing to detect syphilis and severe anaemia, and weight/height measurements [5].

The utilisation of maternal health services is a complex phenomenon and is influenced by several factors operating on both the demand and supply sides. Previous studies have provided a multi-level framework to explain factors determining the utilisation of maternal health services [6] and have identified three levels of barriers: the community and household level, the health service delivery level, and the health sector policy and strategic management level [7]. Supply-side barriers operate at the service delivery level and are characteristics of the health system that hinder service uptake [8]; shortages of supplies, drugs, and basic equipment are among these factors. The existing literature mostly identifies the numerous demand-side factors as important barriers to healthcare utilisation in developing countries, but few have addressed supply-side barriers [9].

As reliable information about the availability of drugs, medical equipment, and infrastructure is extremely important, a health facility assessment of first-level care facilities was conducted in nine districts of Punjab province, Pakistan. Six areas were assessed: (1) the condition of buildings of primary healthcare (PHC) facilities (“infrastructure”), (2) the availability and functionality of “equipment” (3), the availability of essential “supplies” and (4) “medicines” for provision of ANC services, (5) the availability of “guidelines”, and , the availability of “infection control items”. We hope that results from this study will assist policymakers to make informed decisions about resource allocations to improve ANC services.

## Methodology

A cross-sectional health facility assessment was conducted between October 2010 and February 2011.

## Sampling

A multi-stage sampling procedure was adopted to select the districts and clusters of health facilities from each district. Details of the sampling methods are presented elsewhere [10]. Briefly, all of the districts in Punjab province were ranked from 1 to 36 on the basis of a composite indicator of sociodemographic and economic development and grouped into three strata: high, medium, and low. A total of nine districts were then selected: districts Gujranwala, Rawalpindi, and Toba Tek Singh from the high stratum; Sargodha, Multan, and Sahiwal from the medium stratum; and Bahawalnagar, Vehari, and Kasur from the low stratum. From each of these districts, a cluster of 19 health facilities (17 Basic Health Units (BHUs) and 2 Rural Health Centres (RHCs)) were randomly selected, making a total of 171 BHUs and RHCs. Health facilities where no health provider was available for interviews on the first visit were replaced (n = 17). RHCs are relatively larger PHC facilities serving a rural population of 10,000–50,000, while BHUs are relatively smaller health facilities providing PHC services to a rural population of 5,000–10,000. One BHU is available up to each ‘Union Council’ level. Union Councils are electoral units in the Punjab consisting of clusters of 2–3 villages or a single large town/village depending upon the size of the population. A uniform package of health services covering the essential package of health services for PHC in Punjab and infrastructure, physical, and human resource requirements are defined for each level of care. Medical Officers manage RHCs/BHUs and ANC services are supposed to be provided by Lady Health Visitors in these facilities [11].

## Data collection

Data relating to the six areas was collected through an objectively developed semi-structured questionnaire, which was pretested in a non-sampled district, Nankana Sahib. The questionnaire was administered through observation, interviews, and examination of facility records by enumerators. For “infrastructure”, data were collected on essential amenities in the health facilities (such as phone, electricity, generator, water-supply, toilet facilities, waiting area, privacy for examination, cleanliness, and general condition of the buildings such as boundary wall, gate, signboards, and the approach road). Essential ANC equipment was assessed, including blood pressure apparatus and haemoglobin meters. Availability of essential medicines for pregnant women, such as iron, folic acid, multivitamin, calcium, antimalarial and antipyretic tablets, and tetanus toxoid vaccines, were included in the assessment.

A total of eight teams undertook data collection, with each team consisting of a team leader/supervisor and two surveyors/interviewers. They were given hands-on training in a 3-day workshop on data collection and management in the field. Field monitoring was carried out by two Regional Coordinators, a Public Health Consultant, and the Principal Investigator. Data validation was carried out by inspecting 10% of records for accuracy.

## Data analysis

For each item in the six areas assessed, univariate analysis described the frequency and percentage of facilities. The facilities that met more than 80% of the required items were counted as “good” (or “high”), those meeting between 60% and 80% as “average” (or “acceptable”), and those meeting less than 60% as “poor” (or “low”). These three categories were generated for ease of interpretation of findings. All analyses were conducted using SPSS software.

## Results

The overall condition of infrastructure was poor in 31% of BHUs. Only 56% were classified as good, requiring no repair, and 40% were considered clean. A functional generator, separate toilets for men and women, and a phone were available in only 1%, 14%, and 49% of BHUs, respectively. In contrast, the overall condition of infrastructure was good in the majority of the RHCs (94%; Table 1).

**Table 1:** **Facility resources of basic health units (BHUs) and rural health centres (RHCs)**

	**Overall**		**RHCs**		**BHUs**	
Number of facilities	171		18		153	
	*n*	%	n	%	n	%
Infrastructure items						
Good general condition (need for repairs and maintenance)	99	58	13	72	86	56
Cleanliness (good)	68	40	11	61	57	37
Phone (functional)	91	53	16	89	75	49
Electricity (available)	163	95	18	100	145	95
Electricity (functional)	160	94	18	100	142	93
Generator available	18	11	15	83	3	2
Generator (functional)	14	8	12	67	2	1
Availability of water supply	165	96	18	100	147	96
Availability of toilet	151	88	18	100	133	87
Separate toilets for males and females	35	20	13	72	22	14
Waiting area for patients	169	99	18	100	151	99
Privacy for examination of client/pregnant women	157	92	17	94	140	92
Ranking of facilities by availability of infrastructure (no items available/total items in list) × 100)						
Good condition >80 %	49	29	17	94	32	21
Average condition 60–80 %	74	43	1	6	73	48
Poor condition <60 %	48	28	–	–	48	31
Equipment items						
Weighing machine available	139	81	14	78	125	82
Weighing machine functional	130	76	13	72	117	76
Height meter available	28	16	6	33	22	14
Height meter functional	27	16	6	33	21	14
Thermometer available	142	83	15	83	127	83
Blood pressure apparatus available	164	96	18	100	146	95
Blood pressure apparatus functional	161	94	18	100	143	93
Stethoscope available	164	96	18	100	146	95
Stethoscope functional	163	95	18	100	145	95
Fetal stethoscope available	121	71	18	100	103	67
Exam couch available	160	94	18	100	142	93
Refrigerator available	151	88	18	100	133	87
Refrigerator functional	141	82	18	100	123	80
Availability of vaccine carrier	160	94	18	100	142	93
Vaccine carrier functional	151	88	16	89	135	88
Syringe cutter available	148	87	15	83	133	87
Syringe cutter functional	144	84	14	78	130	85
Haemoglobin meter available	68	40	18	100	50	33
Haemoglobin meter functional	54	32	18	100	36	24
Microscope available	68	40	18	100	50	33
Microscope functional	41	24	18	100	23	15
Sterilizer available	132	77	17	94	115	75
Sterilizer functional	97	57	14	78	83	54
Sharps container available	85	50	12	67	73	48
Ranking of facilities by availability of equipment(no items available/total items in list) × 100)						
Good >80%	60	35	15	83	45	29
Average 60–80%	84	49	3	17	81	53
Poor <60%	27	16	–	–	27	18
Supplies items						
Antenatal care cards	131	77	16	89	115	75
Maternal health register	167	98	18	100	149	97
Health education material	127	74	14	78	113	74
Disposable syringes available	152	89	18	100	134	88
Haemoglobin reagents available	41	24	16	89	25	16
Benedict solution available	17	10	14	78	3	2
Blood sugar testing strips available	94	55	13	72	81	53
Urine strips for albumin available	15	9	13	72	2	1
Slides for malaria parasites available	124	73	18	100	106	69
Disinfectant available	122	71	17	94	105	69
Latex gloves available	127	74	16	89	111	73
5-mL disposable syringe available	128	75	16	89	112	73
Branula available	102	60	16	89	86	56
Soap available	139	81	15	83	124	81
Ranking of facilities by availability of supplies (no items available/total items in list) × 100)						
Adequate supplies >80%	54	32	17	94	37	24
Acceptable supplies 60–80%	105	61	1	6	104	68
Poor supplies <60%	12	7	–	–	12	8
Drugs list						
Tetanus toxoid vaccine	156	91	18	100	138	90
Iron tablets	144	84	17	94	127	83
Folic acid tablets	142	83	17	94	125	82
Antimalarial tablets	144	84	14	78	130	85
Antipyretics	152	89	17	94	135	88
Calcium tablets	53	31	9	50	44	29
Multivitamins	106	62	16	89	90	59
Ranking of facilities by availability of drugs (no items available/total items in list) × 100)						
Adequate drug supply >80%	90	53	13	72	77	50
Acceptable drug supply 60–80%	55	32	5	28	50	33
Poor drug supply <60%	26	15	–	–	26	17
Availability and display of guidelines and protocols						
Protocol available	129	75	16	89	113	74
Protocol displayed	125	73	16	89	109	71
Infection control items						
Disinfectant available	122	71	17	94	105	69
Latex gloves available	127	74	16	89	111	73
Sharps container available	85	50	12	67	73	48
5-mL disposable syringe available	128	75	16	89	112	73
Branula available	102	60	16	89	86	56
Soap available	139	81	15	83	124	81
Ranking of facilities by availability of infection control (no items available/total items in list) × 100)						
Good infection control >80%	93	54	15	83	78	51
Acceptable control 60–80%	55	32	3	17	52	34
Poor control <60%	23	13	–	–	23	15

The availability and functionality of the equipment was “low” in 18% of BHUs. Some of the essential equipment, such as height meters, haemoglobin meters, and microscopes, were available in less than 50% of BHUs. A fetal stethoscope, the most essential equipment for ANC services, was available only in 67% of BHUs. RHCs fared better in terms of the availability and functionality of equipment with no RHCs considered “poor” and 83% considered “good”.

The availability of supply items was low in 8% of BHUs; the availability of some essential supply items, such as urine strips for albumin and Benedict’s solution, were particularly low as they were available at less than 5% of BHUs. Haemoglobin reagent and blood sugar testing strips were available in 16% and 53% of BHUs. In contrast, the availability of supplies was high in 94% of RHCs.

The availability of medicines was low in 17% of BHUs. Calcium tablets and multivitamins were deficient in 71% and 41% of BHUs, respectively. The majority (72%) of RHCs had high availability with adequate medicine supplies but the remaining 28% had room for improvement.

Service delivery protocols were available in only 75% of surveyed facilities (74% in BHUs and 89% in RHCs) and similar proportions had them displayed in the facility.

The availability of infection control items was low in 15% of BHUs while all RHCs had adequate or high availability; indeed, 83% of RHCs had high availability scoring above 80%. The availability of sharps containers was particularly low (67% in RHCs and 48% in BHUs). A steriliser, essential equipment for infection control measures, was found to be functional in 54% of the BHUs.

## Discussion

### Key findings

The current study focused on facility resources, in particular infrastructure and availability of equipment, supplies, drugs, and service protocols, for the provision of ANC. A large majority (80–95%) of RHCs were considered “good” in terms of infrastructure or had a high availability of equipment, supplies, and infection control items. The availability of medicines could be improved in RHCs, as only 28% had “acceptable” availability. BHUs fared poorly in comparison to RHCs in all the six areas. In particular, nearly a third of BHUs had “poor” infrastructure and between 8% and 20% had “low” availability of equipment, drugs, and infection control items.

### Comparisons to other studies

The present findings are consistent with those from two national health facility assessments conducted by the Technical Resource Facility in 2012 [12] and by the Pakistan Initiative of Mothers and Newborns in 2005 [13]; however, in some areas, the findings are even more dismal. The deficiencies observed in health facilities in Punjab as reported by the Technical Resource Facility were comparatively less than the values reported herein, with only 7% of outpatient departments and Lady Health Visitors’ rooms requiring extensive repair works. However, only about half of BHUs (237 out of 493) were equipped with more than 75% of the necessary supplies and 33% had more than 75% of the required drugs. With regards to RHCs, 96% (280 out of 291) had more than 50% of the supply items and 95% had at least 75% of the vaccine items available. Over 95% of the RHCs in Punjab had outpatient departments that were classified as being in a good condition. The majority of the RHCs (280 out of 291) had more than 50% of the required supplies, with 95% of RHCs having more than 75% of the assessed vaccines. The Pakistan Initiative of Mothers and Newborns study also showed that essential drugs were not available in most of the BHUs or Mother and Child Health Centres across the nation, and reported that a foetal stethoscope was available in only 39% of these facilities [13]. In another health facility assessment conducted by the Free and Fair Election Network Governance Monitors in 2010, the buildings of 8% of the monitored RHCs in Punjab were in decrepit condition [14], lacking cleanliness and basic hygiene. Further, almost 11% of the monitored RHCs in Punjab did not have latrines or had latrines without running water.

### Policy implications

The findings of the present study, along with whose of previous studies [12-14], has clearly highlighted the poor status of infrastructure and equipment and the low availability of supplies and medicine in Punjab province health facilities, and in particular BHUs. Based on these findings, we call for urgent increases to resource allocation for infrastructure development and maintenance of rural health facilities. Reforms of existing policies on health facility infrastructure and resource management may be required.

### Further research

Many studies have described severe shortages of essential supplies, medications, and human resources. Further research may be needed to understand how these observed supply-side bottlenecks affect different aspects of service provision [1].

## Conclusion

Gaps have been identified in all input modalities of the ANC delivery system in Pakistan. Resources must be diverted to PHC facilities, in particular BHUs, to strengthen facility infrastructure and the provision of equipment and supplies.

## Abbreviations

ANC: Antenatal care
BHUs: Basic Health Units
PHC: Primary healthcare
RHCs: Rural health centres
